# Supplementary material for: Timely epidemic monitoring in the presence of reporting delays: anticipating the COVID-19 surge in New York City, September 2020
Source: BMC Public Health. 2022 May 2;22:871. doi: 10.1186/s12889-022-13286-7 (PMC9058738; doi:10.1186/s12889-022-13286-7)
Supplement: Supplementary file 1 — Additional file 1: Fig. A. Estimated Cumulative Distribution of COVID-19 Reporting Delays, New York City, June 21 – August 1 (Green) and August 15 – September 26, 2020 (Purple). During June 21 – August 1, an estimated 65.2% of cases were reported within 5 days of the date when the diagnostic test was performed. During August 15 – September 26, this proportion had increased to 87.0%. The mean reporting delay was 4.96 days during June 21 – August 1 and 3.31 days during August 15 – September 26. Thereafter, the cumulative distribution remained relatively stable. During September 8 – October 20, an estimated 88.6% of cases were reported within 5 days, while the mean reporting delay was 3.21 days. (Results not shown.). Fig. B. Reported and Projected COVID-19 Diagnoses, New York City, September 6–26, 2020, Including 95% Confidence Intervals. As in Fig. 3 in the main text, reported cases as of September 26 (zt) are indicated by gray-colored datapoints. Projected cases (ζt), based up the estimated distribution ($\hat{\alpha}$α^) of reporting delays, are indicated by the pink datapoints. Computed 95% confidence intervals, based upon the bootstrap method, are also shown for the projected diagnoses from September 20–26. Before September 20, the computed confidence intervals were smaller than the diameters of the datapoints. Fig. C. Projected Daily Case Count as of September 26 Versus Reported Daily Case Count as of November 7. The superimposed 45-degree line indicates equality between the two variables. The arrow shows the data for September 21, where the projected count was 524 and the ultimately reported count was 513. [file 12889_2022_13286_MOESM1_ESM.docx]

# Supplement

## Additional Results

Fig. A compares the estimated cumulative distribution of reporting delays for two non-overlapping time periods: June 21 – August 1; and August 15 – September 26, 2020. Each curve plots the estimated cumulative percentage of diagnosed cases reported up to and including the delay interval measured on the horizontal axis. With $\hat{\alpha}_{u}$ denoting the estimated probability that a diagnosed case will be reported with delay $u$, the figure thus plots $\hat{\Phi}_{u}=\sum_{\nu=0}^{u} \hat{\alpha}_{\nu}$ as a function of the reporting delay time $u$. Comparison of the two cumulative distribution functions shows a significant reduction in the duration of reporting delays during the summer and early fall of 2020. As noted in the figure, the respective estimates for $\hat{\Phi}_{5}$ were: 0.652 for the earlier interval from June 21 – August 1; and 0.870 for the later interval from August 15 – September 26.


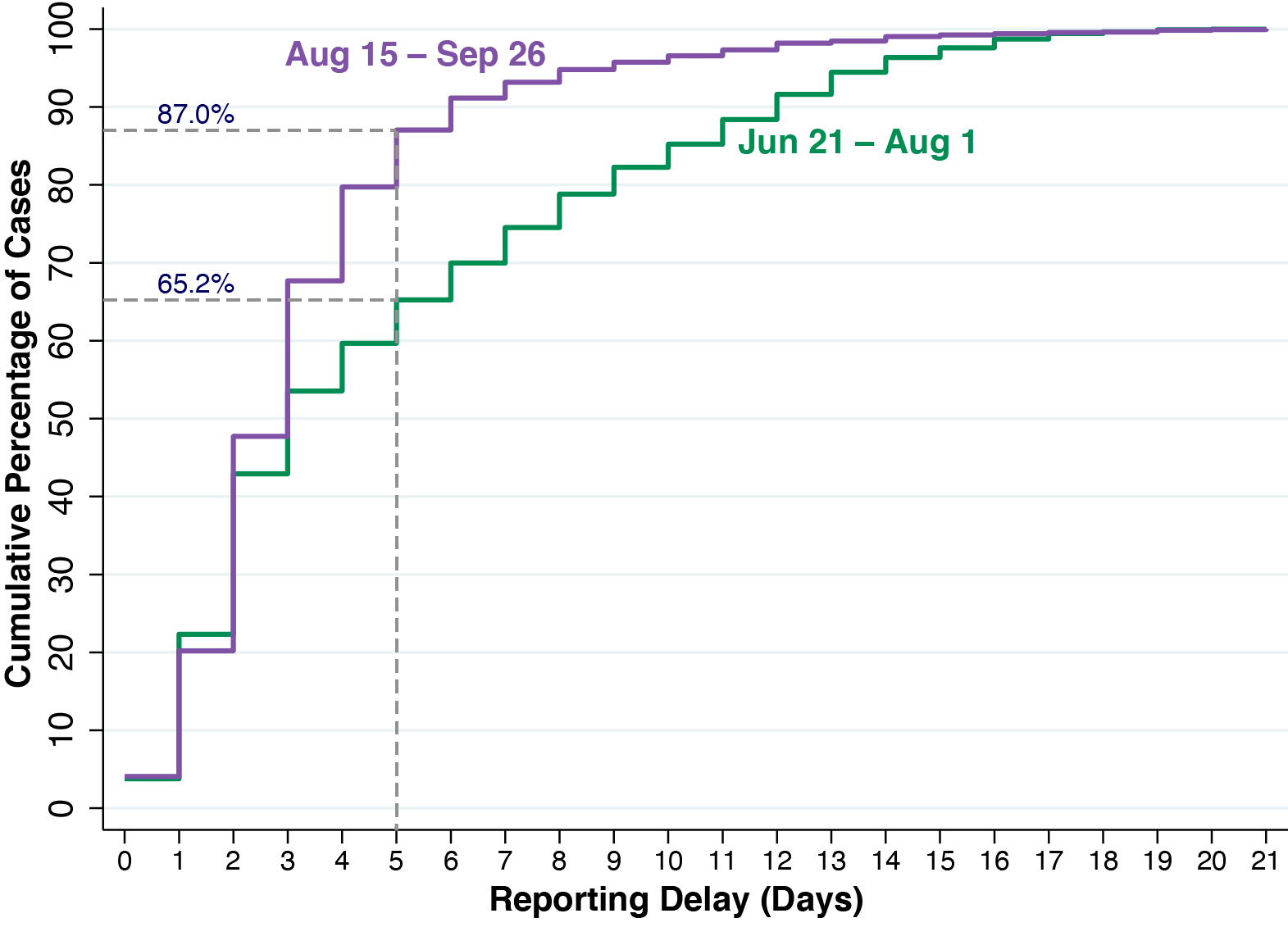


**Fig. A. Estimated Cumulative Distribution of COVID-19 Reporting Delays, New York City, June 21 – August 1 (Green) and August 15 – September 26, 2020 (Purple).** During June 21 – August 1, an estimated 65.2% of cases were reported within 5 days of the date when the diagnostic test was performed. During August 15 – September 26, this proportion had increased to 87.0%. The mean reporting delay was 4.96 days during June 21 – August 1 and 3.31 days during August 15 – September 26. Thereafter, the cumulative distribution remained relatively stable. During September 8 – October 20, an estimated 88.6% of cases were reported within 5 days, while the mean reporting delay was 3.21 days. (Results not shown.)

Fig. B replots the data of Fig. 3 for the date range from September 6 – 26, 2020. As in Fig. 3, the gray datapoints reproduce the observed counts ($z_{t}$) of newly diagnosed COVID-19 cases, while the pink datapoints display projected cases ($\zeta_{t}$), based up the estimated distribution ($\hat{\alpha}$) of reporting delays. Surrounding the pink datapoints from September 20 onward, we show the computed 95% confidence intervals, based upon the bootstrap method. Before September 20, the computed confidence intervals were smaller than the diameters of the datapoints.


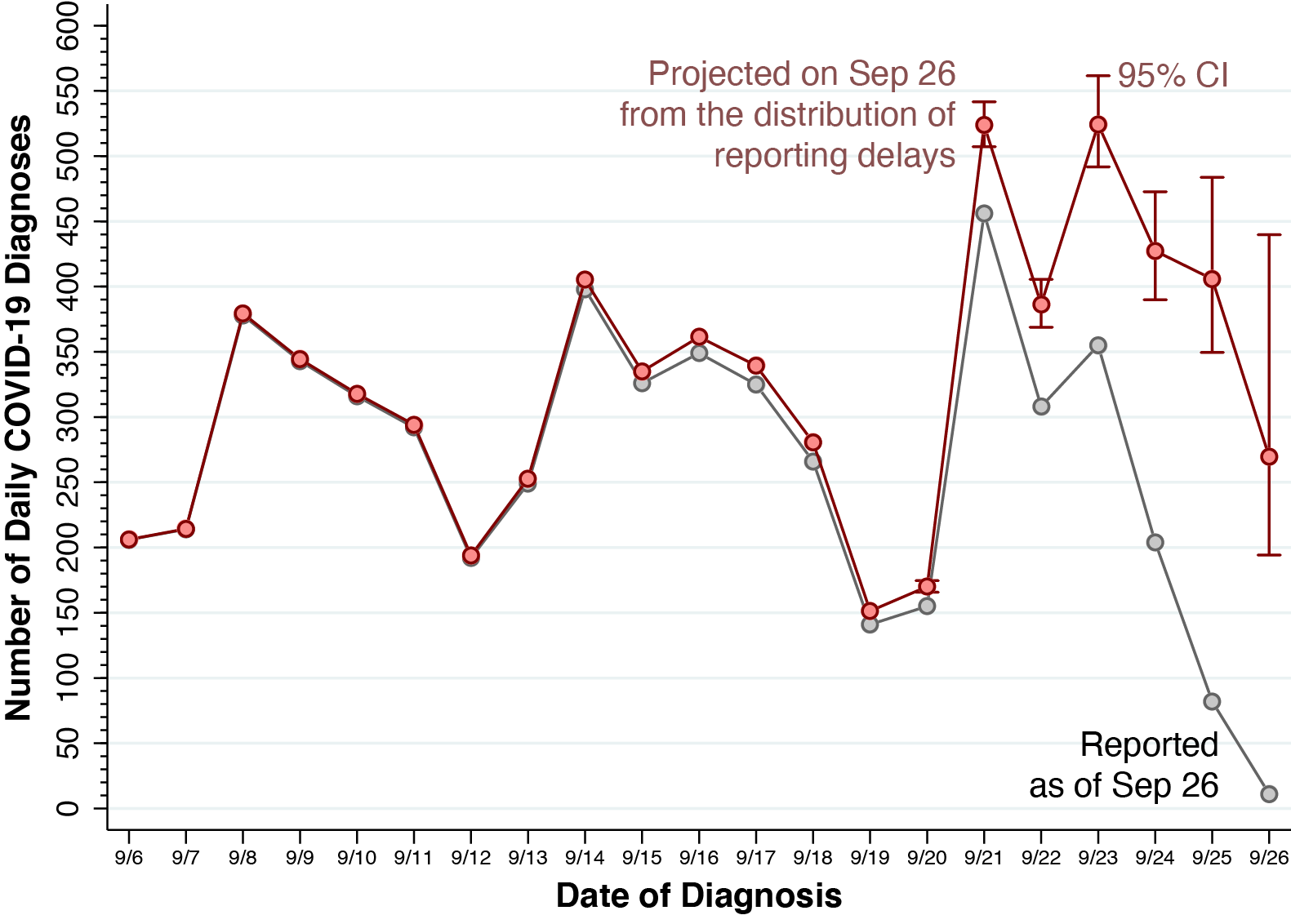


**Fig. B. Reported and Projected COVID-19 Diagnoses, New York City, September 6 – 26, 2020, Including 95% Confidence Intervals.** As in Fig. 3 in the main text, reported cases as of September 26 ($z_{t}$) are indicated by gray-colored datapoints. Projected cases ($\zeta_{t}$), based up the estimated distribution ($\hat{\alpha}$) of reporting delays, are indicated by the pink datapoints. Computed 95% confidence intervals, based upon the bootstrap method, are also shown for the projected diagnoses from September 20 – 26. Before September 20, the computed confidence intervals were smaller than the diameters of the datapoints.

Fig. C plots the projected daily case counts for September 5–26, based upon the data available as of September 26, against the reported daily case counts for the same interval, based upon the data ultimately made available on November 7. Superimposed is a 45-degree line indicating equality between the two variables. The serial correlation coefficient of the projected-versus-reported residuals was 0.266. We could not reject the null hypothesis of serially uncorrelated residuals ($p$ = 0.244).


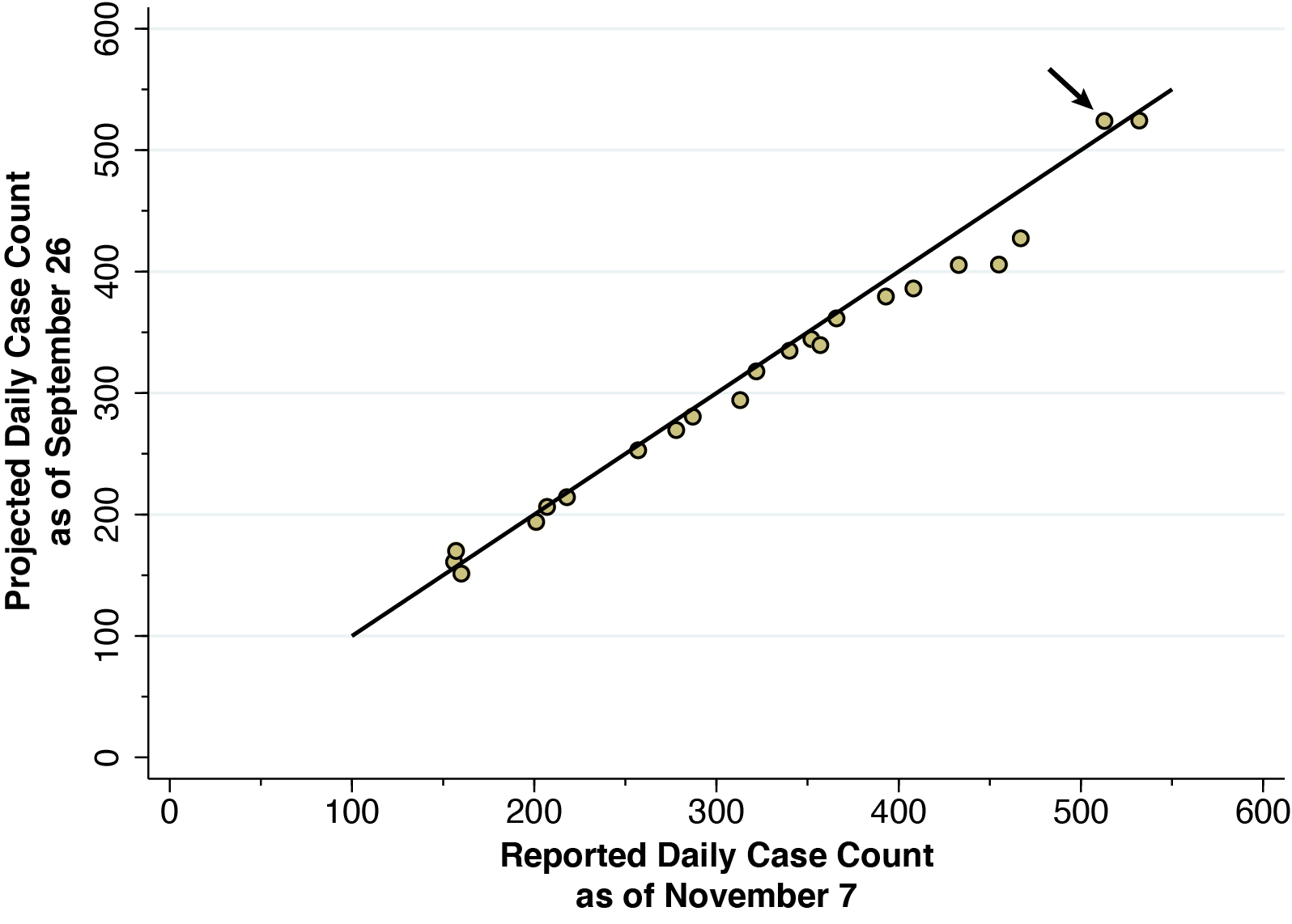


**Fig. C. Projected Daily Case Count as of September 26 Versus Reported Daily Case Count as of November 7.** The superimposed 45-degree line indicates equality between the two variables. The arrow shows the data for September 21, where the projected count was 524 and the ultimately reported count was 513.
